# Physical and Biological Properties of a High-Plasticity Tricalcium Silicate Cement

**DOI:** 10.1155/2018/8063262

**Published:** 2018-11-27

**Authors:** Arthur Dias Galarça, Wellington Luiz de Oliveira Da Rosa, Tiago Machado Da Silva, Giana da Silveira Lima, Neftalí Lenin Villarreal Carreño, Thiago Machado Pereira, Orlando Aguirre Guedes, Alvaro Henrique Borges, Adriana Fernandes da Silva, Evandro Piva

**Affiliations:** ^1^Faculty of Dentistry, Federal University of Pelotas, Pelotas, Brazil; ^2^Department of Restorative Dentistry, Faculty of Dentistry, Federal University of Pelotas, Pelotas, Brazil; ^3^Graduate Program in Materials Science and Engineering, Technology Development Center, Federal University of Pelotas, Pelotas, Brazil; ^4^Department of Oral Sciences, Faculty of Dentistry, University of Cuiaba, Cuiabá, Brazil

## Abstract

**Introduction:**

Mineral Trioxide Aggregate (MTA) is a tricalcium-based silicate, dicalcium silicate matrix. Despite its good biologic properties, some clinicians still claim to have difficulties in handling MTA after its preparation due to its sandy consistency. The aim of the present study was to evaluate the physicochemical properties and cytotoxicity of MTA Repair HP (Angelus, Londrina, PR, Brazil) compared with MTA Angelus (Angelus, Londrina, PR, Brazil).

**Materials and Method:**

The properties assessed were particle size, setting time, flow, film thickness, radiopacity, water solubility, compressive strength, and cytotoxicity. Statistical analysis was performed considering p < 0.05 as statistically significant.

**Results:**

For radiopacity, water absorption and solubility MTA Repair HP were statistically similar to MTA Angelus. The MTA Angelus had statistically different film thickness values, higher than MTA Repair HP (p < 0.05). Besides, MTA Angelus showed a lower and statistically different compressive strength after 28 days than MTA Repair HP (p<0.05). Additionally, MTA Repair HP set more slowly (p < 0.05). Relative to cell viability, MTA Repair HP was statistically similar to MTA Angelus after 24 and 48 h in cell viability.

**Conclusions:**

The MTA Repair HP presented similar cell viability, lower film thickness, higher flow, setting time, and compressive strength values after 28 days than MTA Angelus. In general, the MTA Repair HP presented physicochemical and biological properties similar to the MTA Angelus.

## 1. Introduction

Mineral Trioxide Aggregate (MTA) is a tricalcium-based silicate, dicalcium silicate matrix [1, 2]. Original products contained tricalcium aluminate, tetracalcium aluminoferrite, gypsum, and bismuth oxide [3]. Knowledge about this hydraulic cement increased, and its physical and biological properties expanded [4]. MTA material has excellent biocompatibility and induces tertiary dentin formation after its application in vital pulp therapy [5–7].

The hydration of MTA powder creates a rigid colloidal gel [8]. Despite its good biologic properties, some clinicians still claim to have difficulties in handling this material after its preparation due to its sandy consistency [9, 10]. A commonly encountered problem is that the first MTA products were easily displaced before setting [11]. MTA Repair HP (Angelus, Londrina, PR, Brazil) has been introduced [12] and, according to the manufacturer, this material is easy to manipulate compared to earlier tricalcium-silicate based cements.

According to the MSDS, the MTA Repair HP contains calcium tungstate as radiopacifier in the place of bismuth oxide present in earlier products MTA [12]. Recent studies have shown that MTA Repair HP presents suitable biological properties in human dental pulp stem cells and better push-out bond strength than conventional MTA [1, 9, 10]. However, to the best of our knowledge, no previous study compared MTA Repair HP with a conventional MTA regarding a wide variety of physicochemical properties, such as setting time, flow, film thickness, radiopacity, compressive strength, water solubility, and water absorption, as well as their cytotoxicity effect. Thus, the aim of the present study was to evaluate the physicochemical properties and cytotoxicity of MTA Repair HP (Angelus, Londrina, PR, Brazil) compared with MTA Angelus (Angelus, Londrina, PR, Brazil). The null hypothesis tested was that the MTA Repair HP cement would have physical and biological properties similar to those of MTA Angelus.

## 2. Materials and Methods

The materials evaluated in the present study and their composition are presented in Table 1. All materials were manipulated in accordance with the manufacturer's recommendations.

The setting time, flow, and film thickness were determined in accordance with methods recommended by the International Organization for Standardization (ISO) specification number 6876:2012 [13]. Radiopacity and compressive strength were determined according to the 96-2012 American National Standards Institute/American Dental Association (ANSI/ADA) [14]. Water solubility and water absorption were determined according to the ISO 4049:2000 [15]. Cell viability were determined according to the ISO 10993-5:2009 [16].

### 2.1. Particle Size Analysis

Particle size analysis was performed by laser granulometry (1064, CILAS, Orleans, France). Particle size distributions between 0.04 and 500 *μ*m were characterized. One gram was used for the test. Isopropyl alcohol was used as medium for the samples, with a readout time of 120 s.

### 2.2. Setting Time Analysis

Stainless steel molds with 10 mm inner diameter and 2 mm uniform thickness were fabricated for each material evaluated. After being manipulated, the material was placed in a dental plaster mold kept at a constant temperature of 37°C and 95% air humidity. A Gilmore needle (100 g and 2 mm active tip) was vertically pressed against the horizontal surface of the material to observe indentations. This procedure was repeated at regular intervals of 30 s until no more indentations could be observed on the cement surface. Setting time was defined as the time elapsed from the beginning of the mixture until the time when no more indentations were visible on the cement surface.

### 2.3. Flow Analysis

Using a graduated pipette, 50 *μ*L of the materials was dispensed on a glass plate (40 x 40 mm). The second glass plate was placed on top of the material, followed the 120-g weight after 180 s from the start of mixing. The assembly was left in place for 10 min from the start of mixing, after which the maximum and minimum diameters of the compressed disc of the material were measured using a ruler ±1 mm. Three specimens of each material were made. The mean value of these three specimens was defined as the flow of material.

### 2.4. Film Thickness Analysis

The combined thickness of two glass plates each measuring 5 mm in thickness and having a surface area of 200 mm^2^ was measured with a micrometer (± 1 *μ*m). The materials were manipulated and placed between the 2 glass plates. Ten seconds before the end of the manufacturer's stated working time, the plats were loaded in a loading device (OD57, Odeme, Santa Catarina, Brazil), and a load of 150 N was applied for 10 min. After this time, the thickness of the combined glass plates and material was measured. Three determinations were made for each material evaluated.

### 2.5. Radiopacity Analysis

Five samples of each material (10 mm in diameter and 1 mm thickness) were placed on occlusal radiographic film (Insight, Kodak Company, NY, USA) and radiographed with an X-ray apparatus (Kodak 2200 intraoral X-ray system), operating at 70 kV and 10 mA with exposure time of 0.36 s and a focus-film distance of 30 cm. After processing, optical density or gray tones of images were measured and obtained by means of software ImageJ 1.4 (National Institute of Mental Health, Maryland, USA). The “histogram” was used to measure gray shades, ranging from 0 to 255 pixels. Five points of each specimen were randomly selected to obtain the mean radiopacity value (R) in pixels, which was further transformed into mm/Al according to an aluminum scale (from 0.5 mm to 9.0 mm in equally placed steps of 0.5 mm) also present in the radiograph.

### 2.6. Water Solubility (W_SL_) and Water Absorption (W_SR_) Analyses

Ten specimens of each material were molded (1 mm thickness and 6 mm in diameter). The specimens were weighed after 24 h of setting, after a constant initial mass (m1) was obtained. Then the samples were stored in distilled water and stored in the kiln for one week at 37°C (m2) until a constant final mass (m3) after removal from the solution. The water solubility (W_SL_= [(m1 – m3)/m3] x 100) and sorption (W_SR_= [(m2 – m3)/m3] x 100) were calculated as percentages of the original weight.

### 2.7. Compressive Strength Analysis

Ten specimens of each material were prepared by using a split metal mold measuring 6 mm high and 4 mm in diameter and stored at 37°C until the stipulated period. Specimens were immediately removed from the mold and tested at each time interval (1 h, 24 h, 7 days, and 28 days). The specimens were stored and maintained in 1.0 mL of distilled water until the time of testing, in which the universal testing machine was used (DL500; EMIC, São José dos Pinhais, PR, Brazil) at a crosshead speed of 0.5 mm/min. The maximum load required to fracture each specimen was determined. The compressive strength was recorded in megapascals (MPa), using the equation: *C* = 4*p*/*πd*^2^ where p is the maximum force applied, in Newton, and d is the measured diameter of the specimen, in millimeters.

### 2.8. Cell Viability Analysis

Cell viability analysis was performed using mouse fibroblasts L929 (20 x 10^3^ well^−1^) maintained in Dulbecco's Modified Eagle Medium (DMEM, Lonza, Switzerland). Specimens of each material (n = 6; 5 mm in diameter and 1 mm deep) were placed in 24-well plates with 1 mL of DMEM at 37°C, pH 7.2. After 24 h, 200 *μ*L of eluate from each specimen was transferred to previously prepared 96-well plates and incubated for 24 and 48 h. WST-1 (Roche Applied Science, Germany) was applied to assess cell metabolic function by mitochondrial dehydrogenase activity, and the absorbance at 450 nm was measured via a microplate reader (SpectraMax M5; Molecular Devices, Sunnyvale, CA, USA).

### 2.9. Statistical Analysis

Statistical analysis was performed with SigmaPlot 12 software (Systat Inc, San Jose, CA, USA). For setting time, flow, film thickness, water absorption, and solubility, the data were analyzed using the Student's* t*-test. One-way ANOVA followed by the Tukey test was used for radiopacity. Cell viability was analyzed by the Kruskal-Wallis test, and compressive strength, by the Friedman and Tukey tests. The level of significance was set at p < 0.05.

## 3. Results

### 3.1. Particle Size, Setting Time, Flow, Film Thickness, Radiopacity, Water Absorption (W_SR_), and Solubility (W_SL_)

The mean particle size of the MTA Repair HP was 11.20 *μ*m (2.29-22.40 *μ*m), while that of MTA Angelus was 15.48 *μ*m (5.08-30.08 *μ*m). The MTA Repair HP (13.1 ± 1.0 min) presented a set time higher than MTA Angelus (8.3 ± 0.1 min, p < 0.05). For water absorption, solubility, and radiopacity, MTA Repair HP was statistically similar to MTA Angelus. MTA had a statistically higher (p < 0.05) film thickness (330 ± 80 *μ*m), when compared with MTA Repair HP (194 ± 89 *μ*m). Relative to flow the MTA Angelus (16.08 ± 1.52) was statistically different (p < 0.05) of MTA Repair HP (18.15 ± 1.10), which showed higher values of flow. All results are shown in Table 2.

### 3.2. Compressive Strength

**Figure 1:**
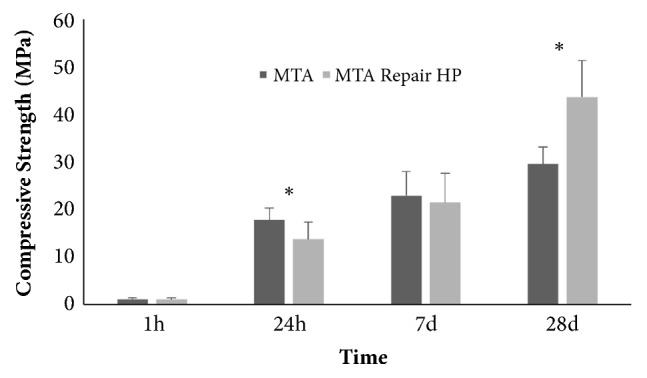
Compressive strength in MPa after 1 and 24 h, 7 and 28 days of storage. In different periods of time, there were statistically significant differences for the same material. *∗* indicates statistically significant differences between the two materials in the same period of time (p<0.05).

Figure 1 shows the results for compressive strength. Both materials increased in compressive strength with time. After 24 h, MTA Angelus compressive strength means significantly higher when compared to MTA Repair HP (p < 0.05). However, after 28 days, MTA Repair HP showed a compressive strength of 43.6 ± 7.7 MPa, a mean that was higher when compared with that of MTA Angelus (30.2 ± 1.8 MPa; p < 0.05).

### 3.3. Cell Viability

**Figure 2:**
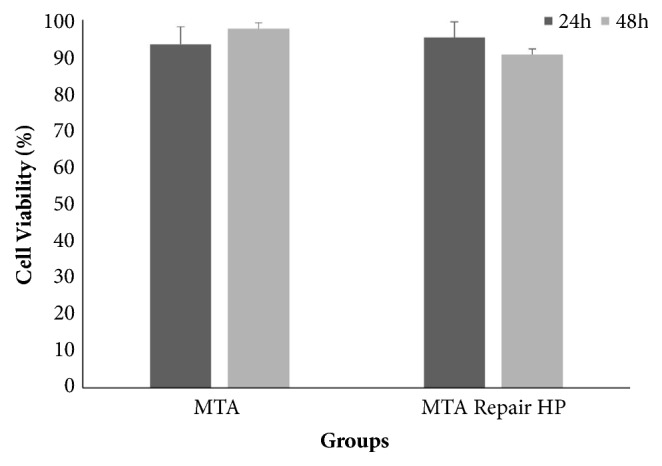
Cell viability and standard deviation (%) of materials evaluated after 24 and 48 h. There were no statistically significant differences among groups and periods of time (p>0.05).

Figure 2 shows the percentage of cell viability assessed after 24 and 48 h. The untreated group (cell control) was considered equal to 100 %. After 24 h, MTA Repair HP showed cell viability of 95.1 %, which was statistically similar (p > 0.05) to that of MTA Angelus (93.3 %). Moreover, after 48 h, MTA Repair HP showed cell viability of 90.7 % that was also statistically similar (p > 0.05) to that of MTA Angelus (97.6 %).

## 4. Discussion

The null hypothesis was partially accepted, since the MTA Repair HP cement demonstrated to be similar to conventional MTA as regards radiopacity, water solubility, water absorption, and cell viability. However, it had higher setting time, flow and compressive strength values after 28 days, and a lower film thickness. The MTA Repair HP cement was developed with the purpose of improving handling properties that could improve material application with an increase in MTA plasticity. In this sense, the results obtained could reflect particularities in the MTA Repair HP composition that were introduced to improve the above-mentioned properties, which will be further discussed.

The longer setting time has traditionally been considered a drawback of ProRoot MTA, with values higher than 40 minutes [11, 17]. Some indications of MTA, such as retrofilling material, require a product with a lower setting time to reduce dislodgement after placement and consequently, solubility and contamination before complete setting [8]. MTA Angelus presents shorter setting time values that ranged from 12 [18] to 24 minutes [4], due to the absence of calcium sulfate in the powder [19]. The present study showed that the MTA Repair HP cement had a longer setting time than MTA Angelus, which could be due to alterations in the radiopacifier component that need to be further investigated. A previous study has demonstrated that presence of calcium tungstate, a radiopacifier, which is present in the MTA Repair HP, has increased the setting time of tricalcium silicate-based cements [4].

Regarding to film thickness, MTA Repair HP had a significantly lower film thickness (but still did not meet the ISO 6876 standard) than MTA Angelus. This property may be explained observations that are in agreement with having a finer particle size. Moreover, the particle size of the new cement was slightly smaller than that of MTA Angelus. Another difference between the composition of MTA Angelus and MTA Repair HP cement was the presence of calcium tungstate as a radiopacifier in the latter, while bismuth oxide is present in MTA Angelus. Ideally, a radiopacifier should only provide the cement with the necessary radiopacity, and should be inert, free of any contaminants, colorless, nontoxic, and added in minimal amounts [7]. Bismuth oxide has been reported to decrease the physicochemical properties of traditional MTA [12, 20, 21] and it is esthetically unsatisfactory when esthetic regions are affected [22, 23]. Considering that the bismuth oxide and calcium tungstate added to MTA are insoluble in water, this may cause greater insolubility of MTA. However, while it may have interfered in solubility [24], the values between MTA and high plasticity material were similar regarding water sorption and solubility.

The use of calcium tungstate, gold and silver/tin alloy [6], and zirconium oxide [25] has been suggested as radiopacifier instead of bismuth oxide [4, 25, 26]. For the MTA Repair HP, the replacement of bismuth oxide by calcium tungstate maintained the material radiopacity higher than 3.0 mmAl as recommended by ISO 6876:2008 [13] and is in accordance with other recent studies that compared MTA Repair HP with MTA Angelus [27]. Recent studies have demonstrated that calcium tungstate improved the physical-chemical, antibacterial, and biological properties of tricalcium silicate-based cements [4, 25]. An in vitro study also showed that MTA Repair HP showed better push-out bond strength than white MTA [10]. Although in our study a lower compressive strength was observed for this cement after 24 h, its values increased with time and were higher than those of MTA Angelus after 28 days. An improvement in compressive strength over time has been reported for MTA [17], which may decrease susceptibility to fracture over the course of time. It has been indicated that layers of linings and the amount of soft carious tissue left behind could affect the strength and longevity of composite restorations [23]. In this sense, a liner material such as MTA with a higher compressive strength could be beneficial since it would withstand greater occlusal stress.

The important property of MTA Angelus of low cytotoxicity has been maintained in MTA Repair HP cement after 24 and 48 h. At present, MTA is considered the material of choice for direct pulp capping [28–30]. High biocompatibility was also observed for both MTAs evaluated in this study, even after 48 h. Another study also showed that MTA Repair HP presents adequate cytocompatibility with human dental pulp stem cells (hDPSCs) [9]. It was also previously demonstrated that MTA Repair HP could promote biological responses in hDPSCs regarding cell proliferation, morphology, migration, and attachment, with the material being cytocompatible [9]. Further evaluation in animal experiments and clinical trials needs to be developed.

## 5. Conclusion

The MTA Repair HP presented similar cell viability, lower film thickness, higher flow, setting time, and compressive strength values after 28 days than MTA Angelus. In general, the MTA Repair HP presented physicochemical and biological properties similar to the MTA Angelus.

## Figures and Tables

**Table 1:** Composition of the tested materials and their manufacturer.

**Materials**	**Composition **
MTA Angelus (Angelus, Londrina, PR, Brazil)	Powder: silicon oxide (SiO_2_), potassium oxide (K_2_O), aluminum oxide (Al_2_O_3_), sodium oxide (Na_2_O), iron oxide (Fe_2_O_3_), calcium oxide (CaO), bismuth oxide (Bi_2_O_3_), magnesium oxide (MgO), insoluble residues of crystalline silica, (K_2_SO_4_), and (Na_2_SO_4_) Liquid: Water

MTA Repair HP (Angelus, Londrina, PR, Brazil)	Powder: tricalcium silicate (3CaO.SiO_2_), dicalcium silicate (2CaO.SiO_2_), tricalcium aluminate (3CaO.Al_2_O_3_), calcium oxide (CaO) and calcium tungstate (CaWO_4_) Liquid: Water and Plasticizer

**Table 2:** Physicochemical properties of MTA and MTA Repair HP.

**Properties**	**MTA**	**MTA Repair HP**
**Particle size (** ***μ*** **m)**	15.48	11.20
**Setting time (min)**	8.3 ± 0.1^a^	13.0 ± 1.0^b^
**Flow (mm)**	16.08 ± 1.52^a^	18.15 ± 1.10^b^
**Film thickness (** ***μ*** **m)**	330 ± 80^a^	194 ± 89^b^
**Radiopacity (mm/Al)**	3.01 ± 0.09^a^	3.04 ± 0.16^a^
**W**_**SR**_ (%)	19.40 ± 2.67^a^	16.32 ± 2.92^a^
**W**_**SL**_ (%)	-3.81 ± 1.25^a^	-2.77 ± 1.18^a^

W_SR_: water absorption; W_SL_: water solubility

Data followed by different letters are statistically different in the same row (p < 0.05).

## Data Availability

The data used to support the findings of this study are available from the corresponding author upon request.
